# Age within schoolyear and attention-deficit hyperactivity disorder in Scotland and Wales

**DOI:** 10.1186/s12889-022-13453-w

**Published:** 2022-05-30

**Authors:** Michael Fleming, Amrita Bandyopadhyay, James S. McLay, David Clark, Albert King, Daniel F. Mackay, Ronan A. Lyons, Kapil Sayal, Sinead Brophy, Jill P. Pell

**Affiliations:** 1 Institute of Health and Wellbeing, University of Glasgow, 1 Lilybank Gardens, Glasgow, G12 8RZ UK; 2 Administrative Data Research Wales, Swansea University Medical School, Swansea, SA2 8PP UK; 3 National Centre for Population Health and Wellbeing Research, Swansea University Medical School, Swansea, SA2 8PP UK; 4 Department of Child Health, University of Aberdeen, Aberdeen, AB25 2ZG UK; 5 Public Health Scotland, Edinburgh, EH12 9EB UK; 6 ScotXed, Scottish Government, Edinburgh, EH6 6QQ UK; 7 Health Data Research UK, Swansea University Medical School, Swansea, SA2 8PP UK; 8 Division of Psychiatry & Applied Psychology, University of Nottingham, Nottingham, NG7 2UH UK

**Keywords:** Attention-deficit hyperactivity disorder, Relative age, Data linkage, Children, Education, School

## Abstract

**Background:**

Previous studies suggest an association between age within schoolyear and attention-deficit hyperactivity disorder (ADHD). Scotland and Wales have different school entry cut-off dates (six months apart) and policies on holding back children. We aim to investigate the association between relative age and treated attention deficit hyperactivity disorder (ADHD) in two countries, accounting for held-back children.

**Methods:**

Routine education and health records of 1,063,256 primary and secondary schoolchildren in Scotland (2009–2013) and Wales (2009–2016) were linked. Logistic regression was used to examine the relationships between age within schoolyear and treated ADHD, adjusting for child, maternity and obstetric confounders.

**Results:**

Amongst children in their expected school year, 8,721 (0.87%) had treated ADHD (Scotland 0.84%; Wales 0.96%). In Wales, ADHD increased with decreasing age (youngest quartile, adjusted OR 1.32, 95% CI 1.19–1.46) but, in Scotland, it did not differ between the youngest and oldest quartiles. Including held-back children in analysis of their expected year, the overall prevalence of treated ADHD was 0.93%, and increased across age quartiles in both countries. More children were held back in Scotland (57,979; 7.66%) than Wales (2,401; 0.78%). Held-back children were more likely to have treated ADHD (Scotland OR 2.18, 95% CI 2.01–2.36; Wales OR 1.70, 95% CI 1.21–2.31) and 81.18% of held-back children would have been in the youngest quartile of their expected year.

**Conclusions:**

Children younger within schoolyear are more likely to be treated for ADHD, suggesting immaturity may influence diagnosis. However, these children are more likely to be held back in countries that permit flexibility, attenuating the relative age effect.

**Supplementary Information:**

The online version contains supplementary material available at 10.1186/s12889-022-13453-w.

## What is known on this subject?

- A number of studies have reported that attention-deficit hyperactivity disorder (ADHD) is associated with younger age within schoolyear (relative age) with most investigators suggesting that this may reflect differential case ascertainment, rather than a causal relationship, because younger children are developmentally less mature than their older classmates with whom their behaviour is being compared. Further research has been recommended on the impact of holding back children on case ascertainment and particularly whether the provision of flexibility in school starting dates masks or reduces the relative age effect.

## What this study adds?

- In Scotland and Wales, cut-off ages for school-entry are six months out of phase. They also employ different approaches to holding back children, with the practice being less restrictive in Scotland. Comparison of the two countries, therefore, provides a useful natural experiment for investigating the relationship between age within schoolyear and ADHD, and investigating whether it is independent of potential confounders and modified by policies on holding back children. Clinicians assessing or treating children and young people for ADHD should be aware that irrespective of the date of cut-off for school entry, children who are younger within their school year are more likely to be treated for ADHD. This trend may be masked in countries with flexible start date policies where younger children with attention or behavioural problems are more likely to be held back a year if the teachers and parents agree that this is in the best interests of the child. Holding back children does not appear to reverse the need for ADHD medication. It is possible that holding back children with ADHD might, nonetheless, improve other outcomes.

## Background

Attention-deficit hyperactivity disorder (ADHD) is a neuro-developmental disorder, characterised by developmentally inappropriate levels of inattention, hyperactivity and impulsiveness. Diagnosis is based on these symptoms affecting the child’s functioning across different settings. Therefore, reports from parents and teachers are considered, in addition to clinical observation and assessment, when making the diagnosis. However, the prevalence of diagnosed or treated ADHD varies greatly between countries, ranging from around 1% in Denmark to over 5% in North America and Iceland [1, 2]. A number of studies have reported that ADHD is associated with younger age within schoolyear (relative age) with most investigators suggesting that this may reflect differential case ascertainment, rather than a causal relationship, because younger children are developmentally less mature than their older classmates with whom their behaviour is being compared [2, 3]. Therefore, younger children may have their immature behaviour misclassified as ADHD (over-ascertainment in younger children) and/or ADHD may not be recognised in some older children who are better able to compensate (more complete ascertainment in younger children). Evidence in support of more complete ascertainment or over-ascertainment among younger children, rather than a genuinely higher incidence in younger children, comes from different sources. Firstly, findings of an association with relative age within schoolyear are more consistent in countries with high ADHD prescription rates, such as the USA, Canada, Iceland, Israel and Germany (pooled risk ratio of 1.27) [2]. In contrast, due to very high heterogeneity, a meta-analysis could not be performed on studies conducted in countries with lower prescription rates [2]. Secondly, in USA states with a fixed 1^st^September school entry cut-off date, rates of ADHD treatment for young children differed between the youngest and oldest children (i.e. those born in September and August); these differences were not found in states that applied different cut-off dates [4]. However, in common with the vast majority of the literature [3], both this study and a recent UK study [5] were hampered by being unable to identify children held back a year and therefore misclassifying their relative age using month of birth. lt has been suggested that the association with relative age may, therefore, have been underestimated because of exposure misclassification [3, 6]. Further research has been recommended on the impact of holding back children on case ascertainment and particularly whether the provision of flexibility in school starting dates masks or reduces the relative age effect [3]. Thirdly, there are only very modest differences in the likelihood of ADHD treatment receipt between children who are young and old within their schoolyear in Denmark [7], where there are reported to be tight, age-specific criteria for diagnosing ADHD and there is considerable parental discretion in deciding to hold back children considered too immature to start school [8]. In Denmark, 40% of children born October-December, who would normally be in the youngest quartile in their year, are held back to the next year [7].

It is unclear how these international findings might generalise to countries within the UK, such as Scotland and Wales, where healthcare is provided free of charge at the point of delivery via the National Health Service, there is clear guidance for the diagnosis and treatment of ADHD [9, 10], and diagnosis and treatment rates are low [1]. In Scotland and Wales, cut-off ages for school-entry are six months out of phase. They also employ different approaches to holding back children, with the practice being less restrictive in Scotland [11]. Comparison of the two countries, therefore, provides a useful natural experiment for investigating the relationship between age within schoolyear and ADHD, and investigating whether it is independent of potential confounders and modified by policies on holding back children.

## Methods

The study was conducted across Scotland (population 5.4million) and Wales (population 3.1million). The two countries are six months out of phase in relation to age at school entry. In Scotland, the cut-off date of birth for entry into school is 1^st^ March and in Wales it is 1^st^ September. Both Scotland and Wales have country-wide coverage of routine health and education data that are linkable at individual-level*.* The authors assert that all procedures contributing to this work comply with the ethical standards of the relevant national and institutional committees on human experimentation and with the Helsinki Declaration of 1975, as revised in 2008. NHS ethics approval is not required for secondary analyses of anonymised extracts of routine data. Access to the Welsh data was carried out under Secure Anonymised Information Linkage (SAIL) Databank Information Governance Review Panel (IGRP) approved project *Wales Electronic Cohort for Children (WECC): Phase 4 *(project number 0916). In Scotland, access was approved by the National Health Service Public Benefit and Privacy Panel (reference 1920–0144) and covered by a data processing agreement between Glasgow University and Public Health Scotland and a data sharing agreement between Glasgow University and the education department of the Scottish Government (ScotXed). All Scottish data were linked by the Electronic Data Research and Innovation Service (eDRIS), part of Public Health Scotland.

### Record linkage

In Scotland, the health sector uses a unique identifier, the community health index (CHI), which enables different health databases to be linked to each other, at an individual level, using exact matching. The education sector uses a different unique identifier, the Scottish Candidate Number (SCN), by which different education databases can be linked. We have previously demonstrated that, for singleton children, probabilistic matching of the CHI and SCN, based on date of birth, sex and postcode of residence, is 99% accurate [12]. The linked data were analysed within a secure National Safe Haven. In Wales, data linkage is performed using an anonymised, encrypted NHS number identifier, known as the anonymised linkage field (ALF), which is generated by the trusted third-party NHS Wales Informatics Service (NWIS). The data linkage and analyses were performed within the SAIL Databank platform [13].

### Inclusion and exclusion criteria

The study was restricted to singleton children who attended mainstream primary or secondary schools in Scotland between 2009 and 2013 and in Wales between 2009 and 2016, and who had been born in the same country and could, therefore, be linked to their maternity records. Multiple births and special schools were excluded because, in Scotland, it is not possible to be certain that records of same sex children have been correctly linked and school stage is not recorded for special schools. In Wales, children who did not have general practice (GP) records in SAIL could not be included. Since the focus of this study was the effect of age within school year, we excluded children who had been advanced or held back by one or more school year from the primary analyses. This is because they were likely to be either atypically gifted or struggling with academic work independent of their age within year. Therefore, their inclusion would have introduced bias. In the supplementary analyses, we re-included children who had been held back by one year only because, due to less stringent restrictions in Scotland, the parents of children who would otherwise be among the youngest in their year sometimes elect to hold them back a year at entry into primary or secondary school to optimise their exam grades. Since this is not done in response to concerns about the child’s academic abilities, their inclusion was less likely to introduce bias.

### Databases

In Scotland, the Scottish Morbidity Record maternity database (SMR02) collects data on maternal, obstetric and child factors relating to pregnancy and delivery. The Prescribing Information System (PIS) collects information on all prescriptions dispensed to Scottish residents by community pharmacies or primary care, and includes prescriptions issued in Scotland but dispensed elsewhere in the United Kingdom. The ScotXed school pupil census is conducted annually, in September, by all local authority run primary and secondary schools. In Wales, the study population was derived from the Welsh Demographic Service (WDS) dataset, which is an administrative database of all individuals living in Wales and registered with a GP. Demographic and maternity data were obtained from the WDS and National Community Child Health Database (NCCHD). Medication history was derived from the Welsh Longitudinal General Practice (WLGP) dataset and education records were obtained from the pre-16 years Educational Attainment Dataset.

### Exposure, outcome and confounder variables

Age within school year was defined using month of birth and was categorised into calendar (three month) quartiles. Oldest to youngest age within schoolyear equated to births in March–May, June–August, September–November and December-February in Scotland, and September–November, December-February, March–May and June–August in Wales. The outcome of interest was treated ADHD. In both countries, this was ascertained by receipt of one or more medication licensed solely for the treatment of ADHD defined as: methylphenidate hydrochloride, dexamfetamine sulphate, atomoxetine or lisdexamfetamine dimesylate. The potential confounders included child (sex, area-based socioeconomic deprivation), maternal (smoking during pregnancy, age at delivery, parity) and obstetric (gestation at delivery, sex-gestation-specific birth weight centile, caesarean delivery, 5-min APGAR score) factors. Area-based socioeconomic deprivation was measured using the Scottish Index of Multiple Deprivation (SIMD) and Welsh Index of Multiple Deprivation (WIMD) in Scotland and Wales respectively. Both were categorised into general population quintiles for the respective countries.

### Statistical analyses

The characteristics of children with and without ADHD were compared using chi-square tests and chi-square tests for trend for categorical and ordinal data respectively. Binary logistic regression models were used to examine the association between age within schoolyear and ADHD univariately, adjusted for child confounders and, finally, adjusted for all confounders. In the supplementary analyses, the characteristics of children who had been held back one year were compared with children who were in their expected year using chi-square tests and chi-square tests for trend for categorical and ordinal data respectively. The main unadjusted and adjusted binary logistic regression models were then rerun including children who had been held back one year in addition to those in their expected year. Analyses were undertaken using R v3.3.2 and Stata MP version 14.1.

## Results

For the primary analyses, the study population comprised 1,002,876 singleton children who were in the expected school year for their age and who attended school in the same country in which they were born. Overall, 8,721 (0.87%) were being treated for ADHD: 5,803 (0.84%) of the 699,325 Scottish schoolchildren and 2,918 (0.96%) of the 303,551 Welsh schoolchildren. The prevalence of ADHD was higher in boys, increased with deprivation, maternal smoking during pregnancy and lower maternal age, birth weight and APGAR score, and had a reverse hockey-stick relationship with gestation at delivery (Table 1).

**Table 1:** Characteristics of schoolchildren in their expected school year, by presence or absence of ADHD

	Scotland					Wales					Overall			
	ADHD		No ADHD		*p* value	ADHD		No ADHD		*p* value	ADHD		No ADHD	
	*N* = 5,803		*N* = 693,522			*N* = 2,918		*N* = 300,633			*N* = 8,721		*N* = 994,155	
	n	%	n	%		n	%	n	%		n	%	n	%
Gender					< 0.001^a^					< 0.001^a^				
Male	4,921	84.80	342,819	49.43		2,469	84.61	153,229	50.97		7,390	84.74	496,048	49.90
Female	882	15.20	350,703	50.57		449	15.39	147,404	49.03		1,331	15.26	498,107	50.10
Welsh/Scottish Index of Multiple Deprivation					< 0.001^b^					< 0.001^b^				
1 (Most deprived)	1,863	32.18	157,444	22.73		987	33.82	73,646	24.50		2,850	32.73	231,090	23.27
2	1,457	25.17	139,997	20.22		667	22.86	62,204	20.69		2,124	24.39	202,201	20.36
3	1,015	17.53	133,947	19.34		537	18.40	59,051	19.64		1,552	17.82	192,998	19.43
4	856	14.79	134,187	19.38		398	13.64	48,846	16.25		1,254	14.40	183,033	18.43
5 (Least deprived)	598	10.33	126,962	18.33		329	11.27	56,886	18.92		927	10.65	183,848	18.51
Missing	14		985			0		0			14		985	
Maternal age (years)					< 0.001^b^					< 0.001^b^				
19	1,005	17.32	56,408	8.13		478	16.38	27,893	9.28		1,483	17.00	84,301	8.48
20–24	1,680	28.95	133,302	19.22		910	31.19	67,145	22.34		2,590	29.70	200,447	20.16
25–29	1,562	26.92	204,476	29.48		777	26.63	87,434	29.09		2,339	26.82	291,910	29.37
30–34	1,071	18.46	196,610	28.35		498	17.07	76,870	25.58		1,569	17.99	273,480	27.51
^3^35	485	8.36	102,714	14.81		255	8.74	41,202	13.71		740	8.49	143,916	14.48
Missing	0		12			0		89			0		101	
Maternal smoking					< 0.001^a^					< 0.001^a^				
No	2,673	51.86	446,432	72.65		452	58.25	65,806	79.13		3,125	52.70	512,238	73.42
Yes	2,481	48.14	168,055	27.35		324	41.75	17,352	20.87		2,805	47.30	185,407	26.58
Missing	649		79,035			2,142		217,475			2,791		296,510	
Gestational age (weeks)					< 0.001^b^					< 0.001^b^				
< 28	21	0.36	721	0.10		17	0.58	643	0.21		38	0.44	1,364	0.14
28–34	181	3.12	13,478	1.94		99	3.39	6,823	2.27		280	3.21	20,301	2.04
35–36	315	5.43	23,315	3.36		137	4.69	10,693	3.56		452	5.18	34,008	3.42
37–41	5,088	87.72	630,151	90.93		2,488	85.26	264,048	87.83		7,576	86.90	894,199	89.99
^3^42	195	3.36	25,369	3.66		177	6.07	18,426	6.13		372	4.27	43,795	4.41
Missing	3		488			0		0			3		488	
Birth weight (g)					< 0.001^b^					< 0.001^b^				
£1,000	25	0.43	938	0.14		11	0.38	568	0.19		36	0.41	1,506	0.15
1,001–1,500	3	0.05	192	0.03		20	0.69	1,498	0.50		23	0.26	1,690	0.17
1,501–2,500	466	8.03	35,769	5.16		227	7.81	14,722	4.91		693	7.96	50,491	5.08
2,501–4,000	4,693	80.87	569,582	82.15		2,345	80.72	248,585	82.89		7,038	80.82	818,167	82.37
4,001–4,500	519	8.94	73,333	10.58		253	8.71	29,356	9.79		772	8.87	102,689	10.34
> 4,500	97	1.67	13,496	1.95		49	1.69	5,186	1.73		146	1.68	18,682	1.88
Missing	0		212			13		718			13		930	
5 min Apgar score					< 0.001^b^					< 0.001^b^				
0–3	42	0.74	3,116	0.45		128	7.06	9,641	4.41		170	2.26	12,757	1.41
4–6	77	1.35	6,305	0.92		24	1.32	1,882	0.86		101	1.34	8,187	0.90
7–10	5,595	97.92	677,504	98.63		1,662	91.62	206,996	94.73		7,257	96.40	884,500	97.69
Missing	89		6,597			1,104		82,114			1,193		88,711	
Parity					0.893^a^					< 0.001^a^				
0	2,630	45.73	315,009	45.64		1,134	42.74	114,650	43.87		3,764	44.79	429,659	45.16
^3^1	3,121	54.27	375,160	54.36		1,519	57.26	146,687	56.13		4,640	55.21	521,847	54.84
Missing	52		3,353			265		39,296			317		42,649	
Mode of delivery					0.207^a^					0.025^a^				
Caesarean section	1,132	19.51	139,915	20.17		512	17.55	57,686	19.19		5,183	59.43	611,291	61.49
Other	4,671	80.49	553,605	79.83		2,406	82.45	242,947	80.81		3,538	40.57	382,862	38.51
Missing	0		2			0		0			0		2	

^a^chi square test for association

^b^chi square test for trend

*P* values produced separately as cohorts could not be combined

In Scotland, the prevalence of ADHD increased from the oldest quartile to the second youngest, but then fell in the youngest quartile (Table 2). In Wales, children in the youngest quartile had the highest prevalence of ADHD (Table 2). Differences between Scotland and Wales persisted following adjustment for child, maternal and obstetric confounders (Table 3). In Wales, in the fully adjusted model, the risk of ADHD increased as age decreased over the four quartiles. In Scotland, it increased steadily over the oldest three but the risk of ADHD among children in the lowest age quartile was not significantly higher than in the highest age quartile.

**Table 2:** Breakdown of ADHD by age within school year

	Scotland*			Wales **		
	Month of birth	N	ADHD N (%)	Month of birth	N	ADHD N (%)
1 (oldest)	Mar-May	186,002	1,450 (0.78)	Sept-Nov	76,944	684 (0.89)
2	June-Aug	191,822	1,637 (0.85)	Dec-Feb	74,430	730 (0.98)
3	Sept-Nov	184,751	1,609 (0.87)	Mar-May	75,655	737 (0.97)
4 (youngest)	Dec-Feb	136,750	1,107 (0.81)	June-Aug	76,552	767 (1.00)

^*^Chi squared test for trend *p* = 0.164

^**^Chi squared test for trend *p* = 0.112

**Table 3:** Logistic regression models of the association between age within school year and ADHD

Age category within school year	Model 1			Model 2			Model 3		
	OR	95% CI	*p* value	OR	95% CI	*p* value	OR	95% CI	*p* value
Scotland									
1 (oldest)	1.00			1.00			1.00		
2	1.10	1.02–1.18	0.012	1.11	1.03–1.19	0.004	1.12	1.04–1.21	0.002
3	1.12	1.04–1.20	0.002	1.12	1.04–1.20	0.003	1.13	1.05–1.22	0.001
4 (youngest)	1.04	0.96–1.12	0.343	1.06	0.98–1.15	0.151	1.06	0.98–1.15	0.154
Wales									
1 (oldest)	1.00			1.00			1.00		
2	1.10	0.99—1.23	0.063	1.16	1.04—1.28	0.007	1.15	1.03—1.28	0.01
3	1.10	0.99—1.22	0.083	1.21	1.09—1.35	< 0.001	1.20	1.08—1.34	< 0.001
4 (youngest)	1.13	1.02—1.25	0.022	1.32	1.19—1.47	< 0.001	1.32	1.19—1.46	< 0.001

*OR* Odds ratio, *CI* Confidence interval

Model 1: univariate

Model 2: adjusted for child (sex, age, deprivation quintile) confounders

Model 3; adjusted for above plus, maternal (smoking, age) and obstetric (parity, gestation at delivery, sex- gestation-specific birthweight centile, caesarean section, 5-min Apgar score) confounders

For the supplementary analyses, the study population comprised 1,063,256 children who were either in their expected schoolyear or had been held back a year, of whom 9,897 (0.93%) had treated ADHD. The prevalence of treated ADHD was 0.92% in Scotland and 0.97% in Wales (*p* = 0.012) (Supplementary Table 1). If the children who were held back a year had been in their expected schoolyear, the prevalence of ADHD in Scotland across the four quartiles from oldest to youngest would have been: 0.79%, 0.89%, 0.97% and 1.01% (chi trend, *p* < 0.001).

More children were held back in Scotland than Wales: 57,979 out of 757,304 (7.66%) versus 2,401 out of 305,991 (0.78%) respectively (Supplementary Table 1). Of the 60,380 held back children, 49,017 (81.18%) would have been in the youngest quartile of their expected year, 8,138 (13.48%) in the third oldest, 2,177 (3.81%) in the second oldest, and only 1,078 (1.74%) in the oldest quartile. Children who were held back a year had a two-fold higher prevalence of ADHD treatment: 1.96% in Scotland, 1.71% in Wales, and 1.95% overall (Supplementary Table 1). Held-back children were more likely to be male, affluent, preterm and low birth weight, and less likely to have been born by Caesarean section or have mothers who smoked during pregnancy (Supplementary Table 2). After adjustment for potential confounders, held-back children remained more likely to have treated ADHD: OR 2.18, 95% CI 2.01–2.36 in Scotland and OR 1.70, 95% CI 1.21–2.31 in Wales respectively (Supplementary Table 3).

## Discussion

The prevalence of treated ADHD in Scotland and Wales was comparable to countries such as Denmark and Finland, and lower than the USA [1]. When our analyses included only children who were in their expected school year, younger relative age was associated with higher risk of ADHD in Wales, but not in Scotland. Scottish children were ten times more likely than Welsh children to be held back a year. However, the lower prevalence of ADHD in the lowest age quartile in Scotland was explained by preferential holding back of children who were closer to the cut-off age (who would, otherwise, have been amongst the youngest in the year) and preferential holding back of children with treated ADHD. When the analyses included held-back children, and was based on their expected schoolyear, there was a clear trend, in both countries, between relative age within schoolyear and treated ADHD.

Children who were held back a year differed from their peers in a number of ways and are likely to be a heterogeneous group. They were more likely to have a range of risk factors for ADHD, including male sex, maternal smoking during pregnancy, preterm birth and low birth weight. However, they were also more likely to be from affluent families, suggesting that the preferential take-up of school deferral by some families might be driven by parental worry about perceived relative immaturity. Whilst we were unable to examine the reasons for why children were held back, we assume that it reflects a belief that younger children with behavioural or attention problems may fare badly competing against older, more mature peers and/or might benefit from additional schooling.

Our study had a number of strengths. Firstly, it was large-scale (sample size exceeding one million) and non-selective, in that it covered the whole population of both countries. Secondly, linkage with educational databases enabled us to distinguish which children had been held back. This addressed a major limitation of most previous studies that could not and, therefore, systematically misclassified the relative age of held-back children based on their month of delivery [3, 5, 6, 14, 15]. Thirdly, linkage of education to maternity records enabled us to adjust for maternal and obstetric confounders as well as sociodemographic confounders. Fourthly, inclusion of and comparison between two countries permitted a natural experiment in which we could examine whether the relationship between age within schoolyear and ADHD was influenced by school-entry date cut-off and different approaches to permitting children to be held back a year. ADHD is 60–90% heritable [16]. Expression of candidate genes related to the dopamine system, is modified by exposure to sunlight [17], and their association with ADHD interacts with season of birth [18, 19]. However, our finding that younger children were at higher risk of ADHD in two countries, six months out of phase, demonstrated that the association with relative age is not simply due to confounding by month or season of birth.

A previous UK study of one million children attending school in England/Wales, Scotland or Northern Ireland reported an increased overall risk of ADHD among those in the bottom three-month age group (adjusted HR 1.36, 95% CI 1.28–1.45) [5]. However, the overall finding was dominated by children from England/Wales who accounted for more than 90% of the cohort and had the same school entry date; the authors reported that they were underpowered to comment on the Scottish sub-group. Furthermore, the study was conducted using only primary care records. The authors did not have access to education records and, therefore, could not differentiate children in their expected schoolyear from children who had been held back.

Limitations of our study include the use of medication to identify children with ADHD. Although this may have resulted in failure to ascertain some diagnosed cases, there is no reason to believe that it would introduce a systematic error in relation to month of birth. Our study used data extracted from routine administrative databases, but these undergo regular quality assurance checks. Had the children who were held back a year been in their expected school year, the prevalence of ADHD in Scotland would have followed the same pattern as Wales, increasing across the four quartiles from oldest to youngest. Also, the prevalence of treated ADHD would have been comparable in the two countries. In both countries, delaying school entry by a year was associated with a greater, not lower, likelihood of treatment for ADHD. Therefore, holding back younger children for a year did not appear to reverse their need for ADHD medication. However, we were unable to ascertain at what stage in their education the children were held back to a lower year and, therefore, whether commencement of ADHD treatment preceded or followed their deferral.

In terms of clinical and policy implications, clinicians assessing or treating children and young people for ADHD should be aware that irrespective of the date of cut-off for school entry, children who are younger within their school year are more likely to be treated for ADHD. This trend may be masked in countries with flexible start date policies where younger children with attention or behavioural problems are more likely to be held back a year if the teachers and parents agree that this is in the best interests of the child. Holding back children does not appear to reverse the need for ADHD medication. It is possible that holding back children with ADHD might, nonetheless, improve other outcomes. Further studies are required to determine whether holding back children with ADHD produces other benefits such as improvements in their behavioural and educational outcomes and wellbeing.

## Supplementary Information

**Additional file 1: Table S1.** Breakdown of ADHD by age within school year.

**Additional file 2: Table S2.** Comparison of the characteristics of children held-back one year with those in their expected school year.

**Additional file 3: Table S3.** Logistic regression models of the association between age within school year and ADHD.

## Data Availability

The authors applied for permission to access, link and analyse the Scottish data and undertook mandatory training in data protection, IT security and information governance. All Scottish data were accessed and analysed within a secure national safe haven environment. Therefore, the datasets generated and analysed during the study are not publicly available. The anonymised routinely collected Welsh data accessed in the study were granted via the SAIL databank. The datasets generated and analysed during the current study are available in the SAIL databank repository, https://saildatabank.com/. The availability of the data is subject to request.
