# Supplementary material for: Age within schoolyear and attention-deficit hyperactivity disorder in Scotland and Wales
Source: BMC Public Health. 2022 May 30;22:1070. doi: 10.1186/s12889-022-13453-w (PMC9150337; doi:10.1186/s12889-022-13453-w)
Supplement: Supplementary file 1 — Additional file 1: Table S1. Breakdown of ADHD by age within school year. [file 12889_2022_13453_MOESM1_ESM.docx]

**Supplementary Table 1.** Breakdown of ADHD by age within school year

|  | Scotland | | | Wales | | |
| --- | --- | --- | --- | --- | --- | --- |
|  | Month of birth | N | ADHD  N (%) | Month of birth | N | ADHD  N (%) |
| Held back up to one year | N/A | 57,979 | 1,135 (1.96) | N/A | 2,401 | 41 (1.71) |
| 1 (oldest) | Mar-May | 186,002 | 1,450 (0.78) | Sept-Nov | 76,948 | 684 (0.89) |
| 2 | June-Aug | 191,822 | 1,637 (0.85) | Dec-Feb | 74,438 | 730 (0.98) |
| 3 | Sept-Nov | 184,751 | 1,609 (0.87) | Mar-May | 75,663 | 738 (0.98) |
| 4 (youngest) | Dec-Feb | 136,750 | 1,107 (0.81) | June-Aug | 76,541 | 768 (1.00) |
| Total |  | 757,304 | 6,938 (0.92) |  | 305,991 | 2,961 (0.97) |
|  |  |  |  |  |  |  |

N number; ADHD attention deficit hyperactivity disorder
