# Supplementary material for: Age within schoolyear and attention-deficit hyperactivity disorder in Scotland and Wales
Source: BMC Public Health. 2022 May 30;22:1070. doi: 10.1186/s12889-022-13453-w (PMC9150337; doi:10.1186/s12889-022-13453-w)
Supplement: Supplementary file 2 — Additional file 2: Table S2. Comparison of the characteristics of children held-back one year with those in their expected school year. [file 12889_2022_13453_MOESM2_ESM.docx]

**Supplementary Table 2.** Comparison of the characteristics of children held-back one year with those in their expected school year

|  | Scotland | | | | | Wales | | | | | Overall | | | |
| --- | --- | --- | --- | --- | --- | --- | --- | --- | --- | --- | --- | --- | --- | --- |
|  | Expected year | | Held back | | P value | Expected year | | Held back | | P value | Expected year | | Held back | |
|  | N=699,325 | | N=57,979 | |  | N=303,551 | | N=2,401 | |  | N=1,002,876 | | N=60,380 | |
|  | n | % | n | % |  | n | % | n | % |  | n | % | n | % |
| Gender |  |  |  |  |  |  |  |  |  |  |  |  |  |  |
| Male | 347,740 | 49.73 | 36,813 | 63.49 | <0.001 | 155,698 | 51.29 | 1,272 | 52.98 | <0.001 | 503,438 | 50.20 | 38,085 | 63.08 |
| Female | 351,585 | 50.27 | 21,166 | 36.51 |  | 147,853 | 48.71 | 1,129 | 47.02 |  | 499,438 | 49.80 | 22,295 | 36.92 |
| Welsh/Scottish Index of Multiple Deprivation |  |  |  |  |  |  |  |  |  |  |  |  |  |  |
| 1 (Most deprived) | 159,307 | 22.81 | 10,605 | 18.31 | <0.001 | 74,633 | 24.59 | 542 | 22.57 | <0.001 | 233,940 | 23.35 | 11,147 | 18.48 |
| 2 | 141,454 | 20.26 | 10,206 | 17.63 |  | 62,871 | 20.71 | 475 | 19.78 |  | 204,325 | 20.39 | 10,681 | 17.71 |
| 3 | 134,962 | 19.33 | 11,565 | 19.97 |  | 59,588 | 19.63 | 526 | 21.91 |  | 194,550 | 19.42 | 12,091 | 20.05 |
| 4 | 135,043 | 19.34 | 13,217 | 22.83 |  | 49,244 | 16.22 | 382 | 15.91 |  | 184,287 | 18.39 | 13,599 | 22.55 |
| 5 (Least deprived) | 127,560 | 18.27 | 12,311 | 21.26 |  | 57,215 | 18.85 | 476 | 19.83 |  | 184,775 | 18.44 | 12,787 | 21.20 |
| Missing | 999 |  | 75 |  |  | 0 |  | 0 |  |  | 999 |  | 75 |  |
| Maternal age (years) |  |  |  |  |  |  |  |  |  |  |  |  |  |  |
| 19 | 57,413 | 8.21 | 4,173 | 7.20 | <0.001 | 28,371 | 9.35 | 214 | 8.91 | <0.001 | 85,784 | 8.55 | 4,387 | 7.27 |
| 20-24 | 134,982 | 19.30 | 10,296 | 17.76 |  | 68,055 | 22.43 | 545 | 22.70 |  | 203,037 | 20.25 | 10,841 | 17.95 |
| 25-29 | 206,038 | 29.46 | 16,002 | 27.60 |  | 88,211 | 29.07 | 734 | 30.57 |  | 294,249 | 29.34 | 16,736 | 27.72 |
| 30-34 | 197,681 | 28.27 | 17,128 | 29.54 |  | 77,368 | 25.50 | 580 | 24.16 |  | 275,049 | 27.43 | 17,708 | 29.33 |
| ≥35 | 103,199 | 14.76 | 10,380 | 17.90 |  | 41,457 | 13.66 | 327 | 13.62 |  | 144,656 | 14.43 | 10,707 | 17.73 |
| Missing | 12 |  | 0 |  |  | 89 |  | <10 |  |  | 101 |  | <10 |  |
| Maternal smoking |  |  |  |  |  |  |  |  |  |  |  |  |  |  |
| No | 449,105 | 72.48 | 37,391 | 72.61 | 0.256 | 66,258 | 78.94 | 406 | 78.83 | 0.01 | 515,363 | 73.25 | 37,797 | 72.68 |
| Yes | 170,536 | 27.52 | 14,102 | 27.39 |  | 17,676 | 21.06 | 109 | 21.17 |  | 188,212 | 26.75 | 14,211 | 27.32 |
| Missing | 79,684 |  | 6,486 |  |  | 219,617 |  | 1,886 |  |  | 299,301 |  | 8,372 |  |
| Gestational age (weeks) |  |  |  |  |  |  |  |  |  |  |  |  |  |  |
| <28 | 742 | 0.11 | 308 | 0.53 | <0.001 | 660 | 0.22 | <=10 |  | <0.001 | 1,402 | 0.14 |  |  |
| 28-34 | 13,659 | 1.95 | 2,028 | 3.50 |  | 6,922 | 2.28 | 73 | 3.04 |  | 20,581 | 2.05 | 2,101 | 3.48 |
| 35-36 | 23,630 | 3.38 | 2,365 | 4.08 |  | 10,830 | 3.57 | 100 | 4.16 |  | 34,460 | 3.44 | 2,465 | 4.08 |
| 37-41 | 635,239 | 90.90 | 51,175 | 88.36 |  | 266,536 | 87.81 | 2,044 | 85.13 |  | 901,775 | 89.96 | 53,219 | 88.14 |
| ≥42 | 25,564 | 3.66 | 2,040 | 3.52 |  | 18,603 | 6.13 | 180 | 7.50 |  | 44,167 | 0.00 | 2,220 | 3.68 |
| Missing | 491 |  | 63 |  |  | 0 |  | <=10 |  |  | 491 |  | <501 |  |
| Birth weight (g) |  |  |  |  |  |  |  |  |  |  |  |  |  |  |
| <=1,000 | 963 | 0.14 | 366 | 0.63 | <0.001 | 579 | 0.19 | <=10 |  | <0.001 | 1,542 | 0.15 |  |  |
| 1,001-1,500 | 195 | 0.03 | 45 | 0.08 |  | 1,518 | 0.50 | 20 | 0.83 |  | 1,713 | 0.17 | 65 | 0.11 |
| 1,501-2,500 | 36,235 | 5.18 | 4,534 | 7.82 |  | 14,949 | 4.94 | 133 | 5.54 |  | 51,184 | 5.11 | 4,667 | 7.73 |
| 2,501-4,000 | 574,275 | 82.14 | 46,158 | 79.66 |  | 250,930 | 82.86 | 1,956 | 81.47 |  | 825,205 | 82.36 | 48,114 | 79.69 |
| 4,001-4,500 | 73,852 | 10.56 | 5,776 | 9.97 |  | 29,609 | 9.78 | 233 | 9.70 |  | 103,461 | 10.33 | 6,009 | 9.95 |
| >4,500 | 13,593 | 1.94 | 1,068 | 1.84 |  | 5,235 | 1.73 | 46 | 1.92 |  | 18,828 | 1.88 | 1,114 | 1.84 |
| Missing | 212 |  | 32 |  |  | 731 |  | <10 |  |  | 943 |  | <42 |  |
| 5 minute Apgar score |  |  |  |  |  |  |  |  |  |  |  |  |  |  |
| 0-3 | 3,158 | 0.46 | 406 | 0.71 | <0.001 | 9,769 | 4.43 | 117 | 7.20 | <0.001 | 12,927 | 1.42 | 523 | 0.89 |
| 4-6 | 6,382 | 0.92 | 707 | 1.24 |  | 1,906 | 0.87 | 14 | 0.86 |  | 8,288 | 0.91 | 721 | 1.23 |
| 7-10 | 683,099 | 98.62 | 55,816 | 98.04 |  | 208,658 | 94.70 | 1,493 | 91.93 |  | 891,757 | 97.68 | 57,309 | 97.88 |
| Missing | 6,686 |  | 1,050 |  |  | 83,218 |  | 777 |  |  | 89,904 |  | 1,827 |  |
| Parity |  |  |  |  |  |  |  |  |  |  |  |  |  |  |
| 0 | 317,639 | 45.64 | 24,422 | 42.44 | <0.001 | 115,784 | 43.86 | 886 | 42.43 | <0.001 | 433,423 | 45.15 | 25,308 | 42.44 |
| ≥1 | 378,281 | 54.36 | 33,122 | 57.56 |  | 148,206 | 56.14 | 1,202 | 57.57 |  | 526,487 | 54.85 | 34,324 | 57.56 |
| Missing | 3,405 |  | 435 |  |  | 39,561 |  | 313 |  |  | 42,966 | 4.28 | 748 |  |
| Mode of delivery |  |  |  |  |  |  |  |  |  |  |  |  |  |  |
| Other | 558,276 | 79.83 | 45,248 | 78.04 | <0.001 | 245,353 | 80.83 | 1,991 | 82.92 | <0.001 | 803,629 | 80.13 | 47,239 | 78.24 |
| Caesarean section | 141,047 | 20.17 | 12,731 | 21.96 |  | 58,198 | 19.17 | 410 | 17.08 |  | 199,245 | 19.87 | 13,141 | 21.76 |
| Missing | 2 |  | 0 |  |  | 0 |  | 0 |  |  | 2 |  | 0 |  |

N number; P values obtained using Chi squared test for association or Chi squared test for trend
