# Supplementary material for: Age within schoolyear and attention-deficit hyperactivity disorder in Scotland and Wales
Source: BMC Public Health. 2022 May 30;22:1070. doi: 10.1186/s12889-022-13453-w (PMC9150337; doi:10.1186/s12889-022-13453-w)
Supplement: Supplementary file 3 — Additional file 3: Table S3. Logistic regression models of the association between age within school year and ADHD. [file 12889_2022_13453_MOESM3_ESM.docx]

**Supplementary Table 3.** Logistic regression models of the association between age within school year and ADHD

| Age category | Model 1 | | | Model 2 | | | Model 3 | | |
| --- | --- | --- | --- | --- | --- | --- | --- | --- | --- |
| within school year | OR | 95% CI | p value | OR | 95% CI | p value | OR | 95% CI | p value |
|  |  |  |  |  |  |  |  |  |  |
| Scotland |  | | | | | | | | |
| Held back a year | 2.54 | 2.35-2.75 | <0.001 | 2.24 | 2.07-2.42 | <0.001 | 2.18 | 2.01-2.36 | <0.001 |
| 1 (oldest) | 1.00 |  |  | 1.00 |  |  | 1.00 |  |  |
| 2 | 1.10 | 1.02-1.18 | 0.012 | 1.11 | 1.03-1.19 | 0.004 | 1.12 | 1.04-1.20 | 0.002 |
| 3 | 1.12 | 1.04-1.20 | 0.002 | 1.12 | 1.04-1.20 | 0.003 | 1.13 | 1.05-1.22 | 0.001 |
| 4 (youngest) | 1.04 | 0.96-1.12 | 0.343 | 1.06 | 0.98-1.14 | 0.173 | 1.06 | 0.98-1.14 | 0.177 |
|  |  |  |  |  |  |  |  |  |  |
| Wales |  | | | | | | | | |
| Held back a year | 1.94 | 1.39-2.63 | <0.001 | 1.70 | 1.22-2.31 | 0.001 | 1.70 | 1.21-2.31 | 0.001 |
| 1 (oldest) | 1.00 |  |  | 1.00 |  |  | 1.00 |  |  |
| 2 | 1.10 | 0.99 - 1.23 | 0.063 | 1.16 | 1.04 - 1.28 | 0.007 | 1.15 | 1.03 - 1.28 | 0.01 |
| 3 | 1.10 | 0.99 - 1.22 | 0.083 | 1.21 | 1.09 - 1.35 | <0.001 | 1.20 | 1.08 - 1.34 | <0.001 |
| 4 (youngest) | 1.13 | 1.02 - 1.25 | 0.022 | 1.32 | 1.19 - 1.47 | <0.001 | 1.32 | 1.19 - 1.49 | <0.001 |

OR odds ratio; CI confidence interval

Model 1: univariate

Model 2: adjusted for child (sex, age, deprivation quintile) confounders

Model 3; adjusted for above plus, maternal (smoking, age) and obstetric (parity, gestation at delivery, sex- gestation-specific birthweight centile, caesarean section, 5 minute Apgar score) confounders
